# Identification of RNA binding protein interacting with circular RNA and hub candidate network for hepatocellular carcinoma

**DOI:** 10.18632/aging.203139

**Published:** 2021-06-16

**Authors:** Binglin Cheng, Jingdong Tian, Yuhan Chen

**Affiliations:** 1 The First School of Clinical Medicine, Southern Medical University, Guangzhou, Guangdong Province 510515, China; 2 School of Biomedical Engineering, Xinhua College of Sun Yat-Sen University, Guangzhou, Guangdong Province 510520, China; 3 Department of Radiation Oncology, Nanfang Hospital, Southern Medical University, Guangzhou, Guangdong Province 510515, China

**Keywords:** circular RNA, RNA binding protein, regulatory network, hepatocellular carcinoma

## Abstract

The interaction between RNA binding protein (RBP) and circular RNA (circRNA) is important for the regulation of tumor progression. This study aimed to identify the RBP-circRNA network in hepatocellular carcinoma (HCC). 22 differentially expressed (DE) circRNAs in HCC were screened out from Gene Expression Omnibus (GEO) database and their binding RBPs were predicted by Circular RNA Interactome. Among them, 17 DERBPs, which were commonly dysregulated in HCC from The Clinical Proteomic Tumor Analysis Consortium (CPTAC), The Cancer Genome Atlas (TCGA) and International Cancer Genome Consortium (ICGC) projects, were utilized to construct the RBP-circRNA network. Through survival analysis, we found TARDBP was the only prognostic RBP for HCC in CPTAC, TCGA and ICGC projects. High expression of TARDBP was correlated with high grade, advanced stage and low macrophage infiltration of HCC. Additionally, gene set enrichment analysis showed that dysregulated TARDBP might be involved in some pathways related to the HCC pathogenesis. Therefore, a hub RBP-circRNA network was generated based on TARDBP. RNA immunoprecipitation and RNA pull-down confirmed that hsa_circ_0004913 binds to TARDBP. These findings indicated certain RBP-circRNA regulatory network potentially involved in the pathogenesis of HCC, which provides novel insights into the mechanism study and biomarker identification for HCC.

## INTRODUCTION

Hepatocellular carcinoma (HCC) is most common types of primary liver cancer [1]. Due to the complex etiology of HCC and diverse molecular subtypes detected in individuals, it is pretty hard to make early diagnosis. In consequence, advanced HCC is the main diagnosis of patients and their five-year survival rate is only 10.1% [2]. Thus, there are urgent needs to exploit more effective biomarkers and therapeutic targets for HCC [3].

Circular RNAs (circRNAs), generated by back-splicing from pre-mRNAs, are a special kind of RNAs. Some studies have proved that circRNAs have an impact on a variety of malignant phenotypes? of HCC [4]. And RNA binding proteins (RBPs) are of great importance in RNA dynamics, including subcellular localization, translational efficiency and metabolism [5]. Previous studies have demonstrated that circRNAs can act as sponges of RNA binding protein (RBP), in the meantime RBPs are also able to participate in back-splicing. Therefore, the interaction with RBPs can be also regarded as a crucial element to explore functions of circRNAs [6]. Recent studies have also proved that RBP-circRNA interactions play a significant role in the pathogenesis of cancer. For example, it has been authenticated that circ-DONSON can directly interact with proteins from the NURF chromatin remodeling complex and motivate the transcription of SOX4, which can enhance the occurrence of gastric cancer [7]. However, there are very few studies related to the effects of RBP-circRNA interactions on HCC, which requires more exploration.

In this study, we screened out the differently expressed (DE) circRNA in HCC cases from Gene Expression Omnibus (GEO) database and predicted the RBPs binding to DEcircRNA. After evaluating the expression level of RBPs in HCC from The Clinical Proteomic Tumor Analysis Consortium (CPTAC), International Cancer Genome Consortium (ICGC) and The Cancer Genome Atlas (TCGA) projects, we utilized 17 common DERBPs to construct the RBP-circRNA regulatory network in HCC. Among these RBPs, Tat activating regulatory DNA-binding protein (TARDBP) was the only factor affecting the prognosis of HCC in CPTAC, TCGA and ICGC projects. We further evaluated the expression level of TARDBP in HCC with different clinical traits and immune cell fractions. Gene set enrichment analysis of HCC cases with different level of TARDBP were also assessed. The hub RBP-circRNA network was generated based on TARDBP and the interaction between TARDBP and hsa_circ_0004913 was also experimentally validated. Our findings indicated that certain RBP-circRNA network may be closely related to HCC, which provides ideas for the mechanism study for HCC.

## RESULTS

### Identification of DEcircRNAs and DERBPs in HCC

**Figure 1:**
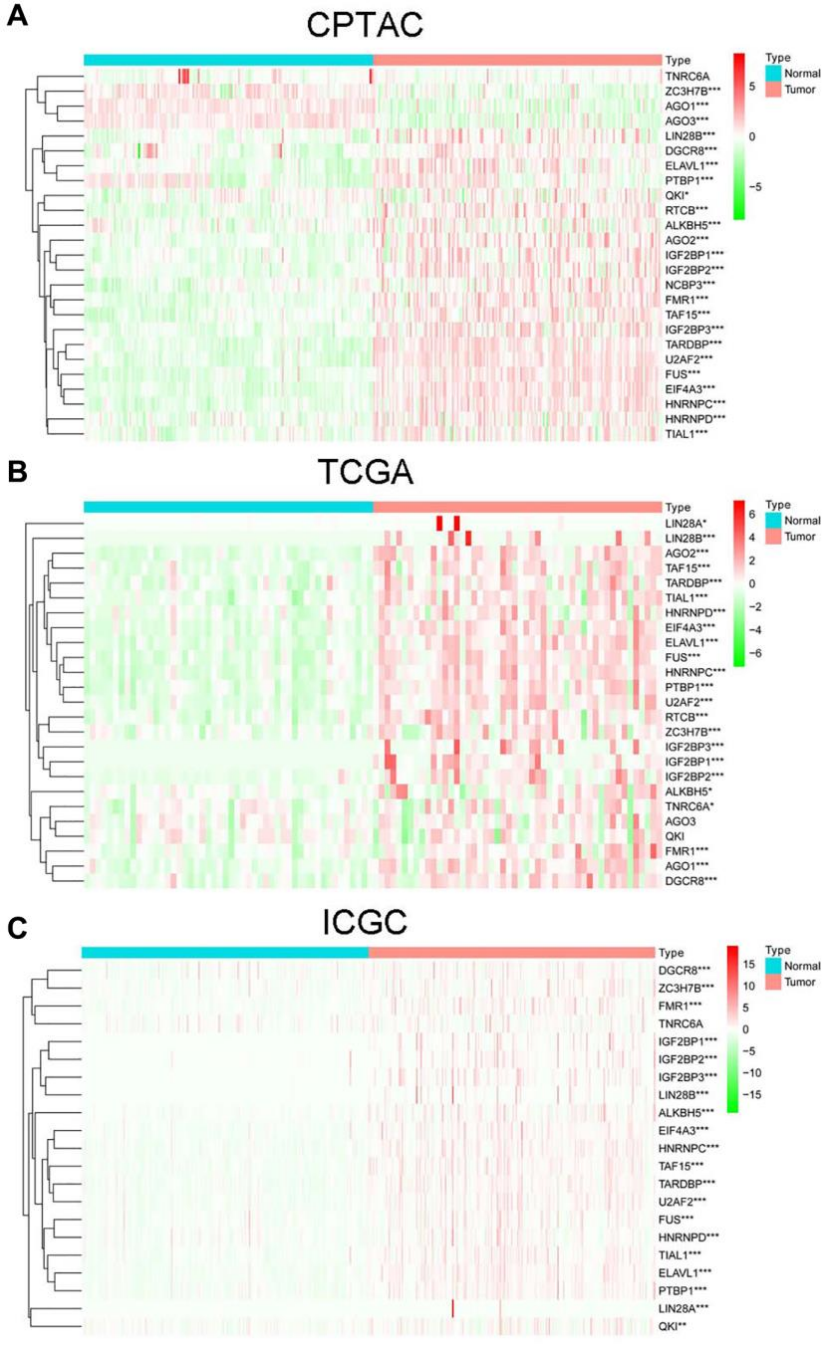
The expression of RBPs in non-tumor and HCC cases from CPTAC (**A**), TCGA (**B**) and ICGC (**C**) projects.

A total of 22 DEcircRNAs (8 up-regulated and 14 down-regulated) were identified by comparing the circRNA microarray data of 15 paired non-tumor and HCC cases from GEO database (Supplementary Table 1). And there were 26 RBPs binding to 22 DEcircRNAs predicted by Circinteractome (Supplementary Table 2). Among them, 25 RBPs were detected in CPTAC project, of which 24 were significantly dysregulated (21 up-regulated and 3 down-regulated) between tumor and normal tissues (Figure 1A). 25 RBPs were detected in TCGA project, of which 23 were significantly dysregulated (23 up-regulated and 0 down-regulated) (Figure 1B). 21 RBPs were detected in ICGC project, of which 20 were significantly dysregulated (20 up-regulated and 0 down-regulated) (Figure 1C). Finally, 17 common DERBPs were obtained after taking the intersection of all up-regulated RBPs.

### Construction of the RBP-circRNAnetwork in HCC

**Figure 2:**
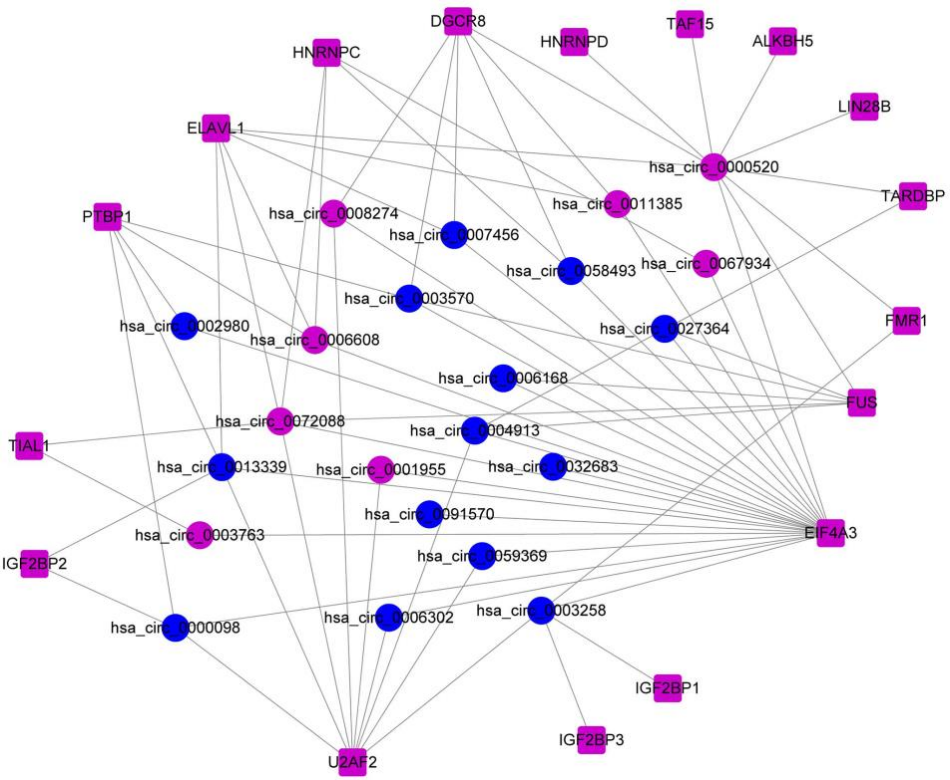
Construction of the RBP-circRNA regulatory network in HCC.

Utilizing 17 common DERBPs and their corresponding DEcircRNAs with biding sites, we constructed the RBP-circRNA regulatory network in HCC. This network contained 72 pairs of RBP-circRNA, including 17 DERBPs and 22 DEcircRNAs (Figure 2).

### Prognostic value and clinical traits correlation analysis of TARDBP in HCC

**Figure 3:**
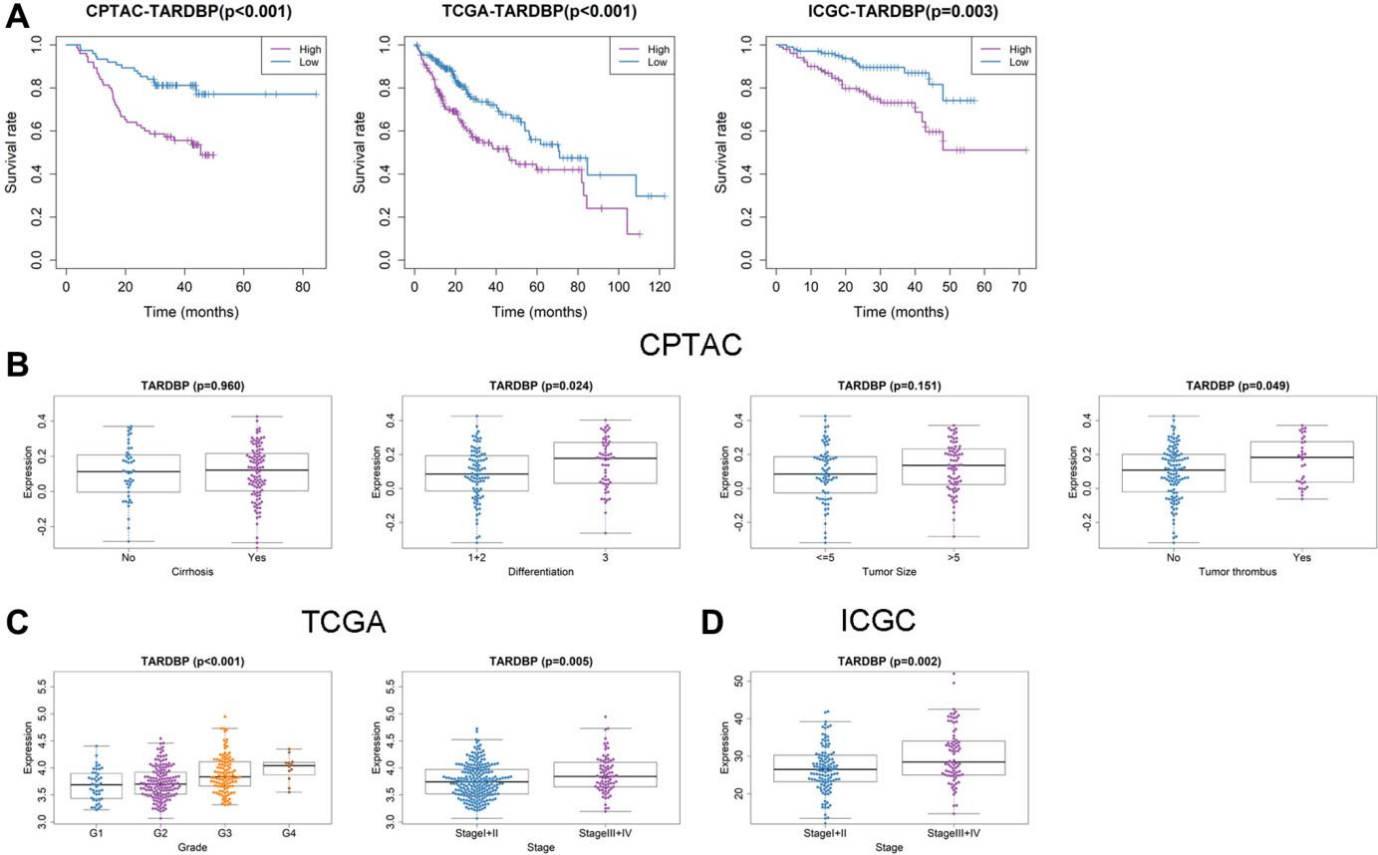
**Prognostic value and clinicopathologic characteristics correlation of TARDBP in HCC.** (**A**) Survival analysis of TARDBP in HCC from CPTAC, TCGA and ICGC projects. (**B**–**D**) Comparison of TARDBP expression level between different clinicopathologic characteristics in CPTAC (**B**), TCGA (**C**) and ICGC (**D**) projects.

**Figure 4:**
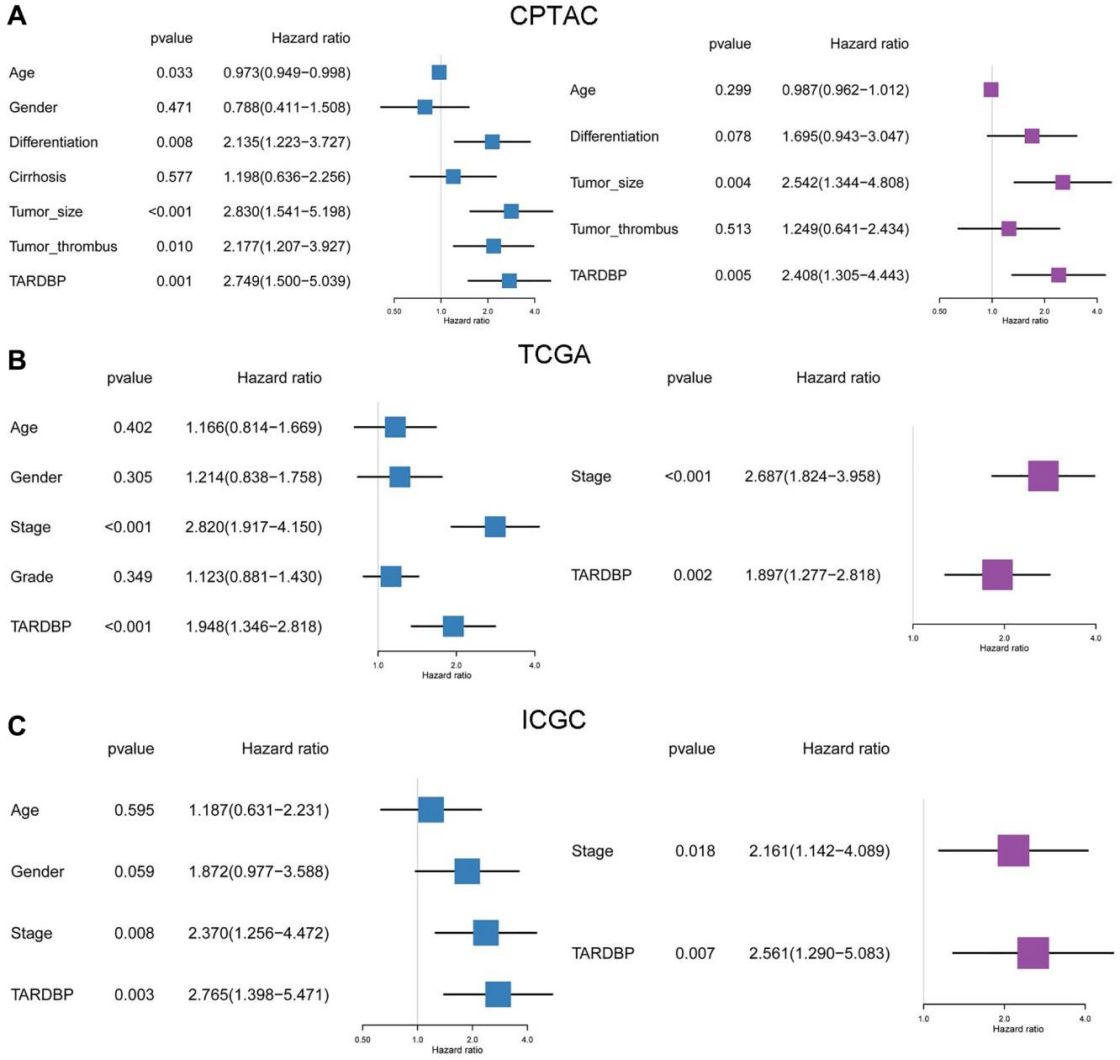
Prognostic analysis of TARDBP and other clinicopathological traits for HCC from CPTAC (**A**), TCGA (**B**) and ICGC (**C**) projects by Cox regression.

In order to screen out the hub RBP, we performed the survival analysis of 17 DERBPs. In CPTAC project, high level of TARDBP or U2AF2 had bad effects on OS of HCC patients (Figure 3A and Supplementary Figure 1). In TCGA project, high expression of LIN28B, EIF4A3, TARDBP, IGF2BP3, PTBP1, DGCR8 or HNRNPC was related to poor OS of HCC cases (Figure 3A and Supplementary Figure 2). While HCC cases with high expression of EIF4A3, IGF2BP3, U2AF2, TARDBP, IGF2BP1, TAF15, HNRNPC, or PTBP1 had shorter OS in ICGC project (Figure 3A and Supplementary Figure 3). After taking intersection, TARDBP was the only prognostic RBP in all three projects. Next, we analyzed the correlation between TARDBP expression and clinical traits. High TARDBP expression was related to high grade of differentiation and more possibilities of tumor thrombus in CPTAC (Figure 3B). Similarly, TARDBP expression was higher in HCC cases with high grade and advanced stage from TCGA (Figure 3C). And in ICGC, high TARDBP expression was also correlated with advanced stage (Figure 3D). Due to the lack of data about pathological grade in ICGC project, the relationships between TARDBP expression and grade could not be fully investigated. Moreover, TARDBP was an independent prognostic factor for HCC cases from CPTAC (Figure 4A), TCGA (Figure 4B) and ICGC (Figure 4C) projects.

### GSEA of HCC cases with different TARDBP expression

**Figure 5:**
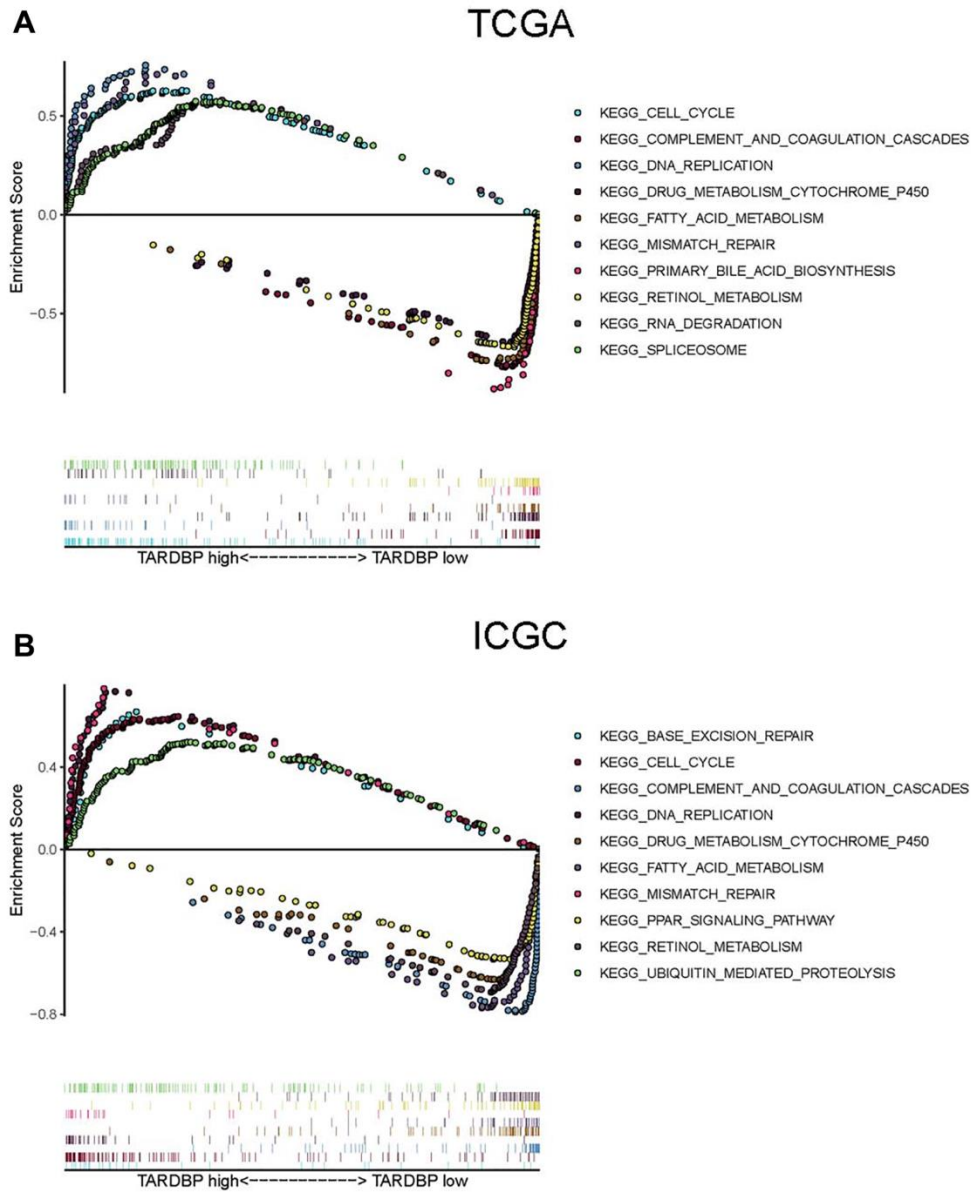
GSEA of HCC cases with TARDBP low- and high-expression in TCGA (**A**) or ICGC (**B**) projects.

To explore the potential function of TARDBP, the HCC cases from ICGC or TCGA were divided into TARDBP low- and high-expression groups. Then potential biological pathways enrichment of two groups was performed by GSEA. We found that TARDBP down-regulated group was enriched in complement and coagulation cascades, retinol metabolism, fatty acid metabolism and drug metabolism cytochrome P450, whereas TARDBP up-regulated group was significantly enriched in the process of cell cycle, DNA replication and mismatch repair in both TCGA (Figure 5A) and ICGC (Figure 5B) projects. In order to verify the results from TCGA and ICGC, we utilized the HCC cases from GSE14520 to perform GSEA. Similarly, TARDBP up- or down-regulated group was significantly enriched in the process of cell cycle or complement and coagulation cascades, respectively (Supplementary Figure 4). It indicated that these two pathways play important roles in the progression of HCC with dysregulated expression of TARDBP.

### High expression of TARDBP is associated with low macrophage fraction in HCC

**Figure 6:**
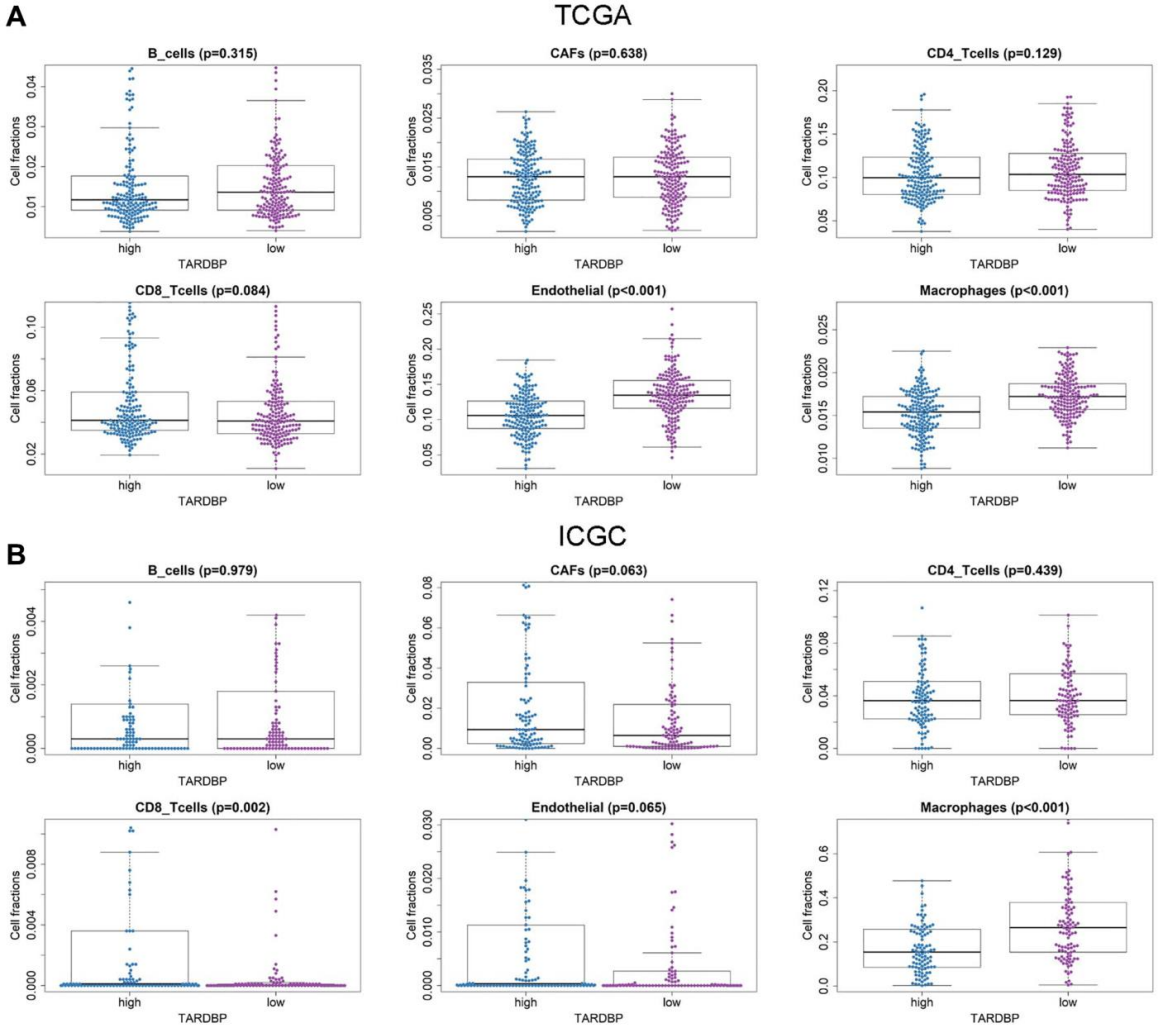
Immune cell fractions of HCC cases with TARDBP low- and high-expression from TCGA (**A**) and ICGC (**B**) projects.

Furthermore, the immune cell fractions of HCC cases with TARDBP low- and high-expression in ICGC and TCGA were analyzed by EPIC application. Among them, fractions of endothelial cells and macrophage were decreased in HCC cases with TARDBP high expression from TCGA (Figure 6A). While TARDBP high expression was detected in HCC cases with low macrophage and high CD8_Tcells fraction from ICGC (Figure 6B). Similar to TCGA results, low fractions of endothelial cells and macrophage were detected in HCC cases with TARDBP high expression from GSE14520 Supplementary (Figure 4B). Notably, high TARDBP expression was accompanied with low macrophage infiltration of HCC in TCGA, ICGC and GSE14520 projects.

### hsa_circ_0004913 binds to TARDBP

**Figure 7:**
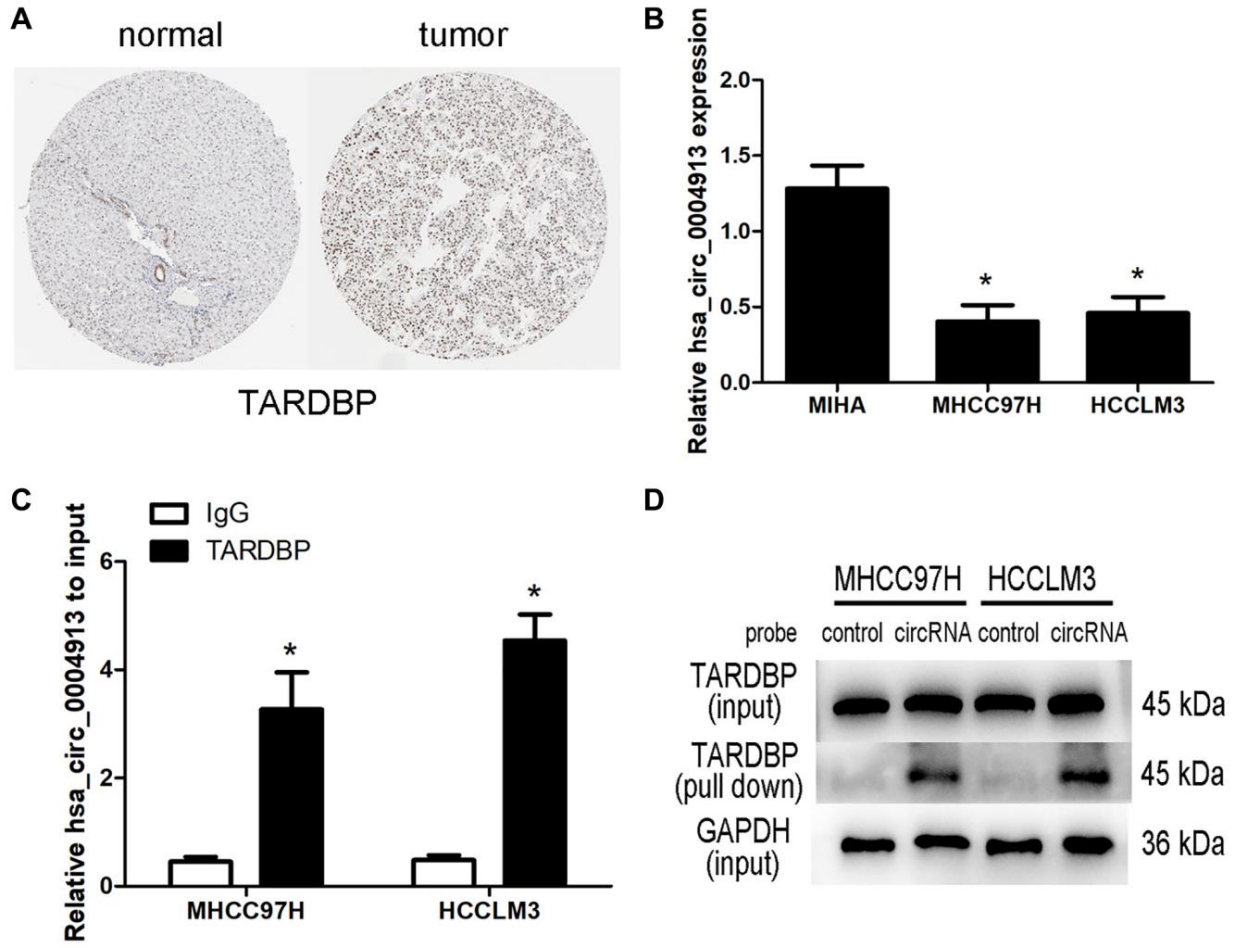
**Analysis of TARDBP- hsa_circ_0004913 network in HCC.** (**A**) The immunohistochemistry staining of TARDBP in normal and HCC tissues from HPA. (**B**) qRT-PCR analysis of hsa_circ_0004913 in MIHA (normal hepatocyte) and HCC cells. *P* < 0.05 vs. MIHA cell. (**C**) RIP analysis of hsa_circ_0004913 enriched in TARDBP IP samples compared with IgG samples. *P* < 0.05 vs. IgG samples. (**D**) RNA pull-down analysis of the binding ability of hsa_circ_0004913 for TARDBP.

Based on the clinical significance of TARDBP, those circRNA binding to TARDBP were extracted to generate a hub RBP-circRNA network and finally two circRNAs, namely hsa_circ_0000520 and hsa_circ_0004913 were included. Among this hub network, TARDBP and hsa_circ_0000520 were up-regulated while hsa_circ_0004913 was down-regulated. The immunohistochemistry staining revealed that TARDBP was higher in HCC than in normal liver tissue from the Human Protein Atlas (HPA, https://www.proteinatlas.org/) project (Figure 7A). A previous study reported that there is no significant difference in hsa_circ_0000520 expression between HCC and adjacent nontumorous tissues [8]. While real-time PCR assay confirmed that hsa_circ_0004913 expression decreased in HCC both in sequencing and GEO database [9]. Thus, we focused on the interaction between hsa_circ_0004913 and TARDBP in HCC. Compared with normal hepatocyte MIHA cells, hsa_circ_0004913 expression was significantly down-regulated in HCC97H and HCCLM3 (Figure 7B). Moreover, RIP assays demonstrated that hsa_circ_0004913 could be enriched in TARDBP IP samples (Figure 7C) and RNA pull down assays also confirmed that hsa_circ_0004913 could bind to TARDBP in HCC cells (Figure 7D), which supports the existence of TARDBP-hsa_circ_0004913 complex.

## DISCUSSION

Previous studies have found that circRNAs are important for the pathogenesis of HCC [10]. According to circRNA microarray data of HCC patients in GEO database, a total of 22 DEcircRNAs were identified. Among them, hsa_circ_0067934 could sponge miR-1324 to activate the FZD5/Wnt/β-catenin pathway, and then promote the proliferation, invasion and migration of HCC cells [11]. In addition, hsa_circ_0091570 modulate the expression of ISM1 by sponging miR-1307 to exert tumor-suppressing capacity in the mouse xenograft model of HCC [12]. Apart from the reported mechanism above, there are very few articles at present discussing their effects on HCC from the interaction between circRNAs and RBPs. Up to now, it has been identified that circRNA acts as a protein scaffolding or a sponge for RBP to modulate the activity of proteins and their downstream targets [13]. For instance, circZKSCAN1 competes with CCAR1 mRNA by binding to FMRP, inhibiting the transcriptional activity of Wnt signaling and subsequently restraining the growth of HCC stem cells [14]. Therefore, we hypothesized that some DEcircRNAs may also exert effects on HCC by interacting with RBPs and thus explored the potential RBP-circRNA regulatory network in HCC.

By using Circinteractome, we found that 26 RBPs bind to these DEcircRNAs and finally 17 common DERBPs were utilized to construct the RBP-circRNA regulatory network. Five DERBPs (TARDBP, EIF4A3, HNRNPC, IGF2BP3 and PTBP1) had impacts on survival in both TCGA and ICGC projects. It was reported that some DERBPs participate in the pathogenesis of HCC. For example, IGF2BP3 increases the steady-state level of Linc01138 RNA and contributes to colony formation and migration of HCC cells [15]. Moreover, PTBP1 expedites the metastasis and invasion of HCC cells by inhibiting the alternative splicing of Axl exon 10 and competing with U2AF2 via binding to the polypyrimidine sequence on Axl-intron 9 [16]. Furthermore, it has been identified that EIF4A3 is highly expressed in HCC, which also contributes to the metastasis and poor prognosis of HCC [17, 18]. Especially, some circRNAs binding to EIF4A3 has also been illustrated to participate in the pathogenesis of HCC. For example, overexpression of hsa_circ_0007456 makes HCC cells more sensitive to NK cytolysis, aiding in the inhibition of tumor immune evasion and tumorigenesis of HCC [19]. Based on their opposite effects on HCC, we deduced that overexpression of hsa_circ_0007456 may act as sponge of EIF4A3, suppressing the role of EIF4A3 in invasion of HCC. Moreover, hsa_circ_0003763 is up-regulated in HCC tissues and facilitate the migration and invasion of HCC cells [20]. According to the cancer-promoting effects of hsa_circ_0003763 and EIF4A3, we speculated that overexpression of hsa_circ_0003763 may function as a dynamic scaffolding molecule to enhance the stability of EIF4A3, promoting EIF4A3-associated tumor metastasis. However, among the DERBPs, only two DERBPs were correlated with OS in CPTAC project. The inconsistent results may be ascribed to the different functions of mRNA and protein during the process of diseases. Beside direct translation into the corresponding protein, some RNA may also affect the post-transcriptional regulation of some other RNAs. The specific reason of this inconsistent result remains to be elucidated in the future studies.

TARDBP was the only RBP with prognostic significance of HCC cases from TCGA, ICGC and CPTAC projects. TARDBP was also an independent prognostic factor for HCC after multivariate Cox regression analysis. High expression of TARDBP was found in HCC case with advanced TNM stage and high pathological grade, which may facilitate it as a biomarker for diagnosis and staging of HCC. Previous research has shown that TARDBP could suppress the expression of phosphofructokinase (PFKP) by inhibiting miR-520 [21]. PFKP is the rate-limiting enzyme for glycolysis [22] and silencing of TARDBP expression results in inhibition of glucose metabolism and HCC proliferation [21]. Although the mechanism of TARDBP on HCC cells has been demonstrated, there is still no article about the relationship between TARDBP and immune cell fractions of HCC patients. It has been reported that a high macrophage fraction can cause better OS of HCC [23]. In this study, TARDBP high expression was correlated with reduced macrophage fraction, which inspires us to suppose that TARDBP exerts influence on the progression of HCC by affecting the activity of macrophage, but the specific mechanism still needs to be discovered. What’s more, by GSEA, we found several important pathways related to high expression of TARDBP, including cell cycle, mismatch repair and DNA replication. These pathways have been confirmed to be concerned in the occurrence and development of HCC [24–26]. And there were also 4 pathways correlated with low expression of TARDBP, including fatty acid metabolism, drug metabolism cytochrome p450, retinol metabolism and complement and coagulation cascades, which were also found to be involved in the progress of HCC [27–30]. These results indicated that TARDBP related circRNA regulatory networks may be potentially involved in the pathogenesis of HCC through participating in some important biological process, such as cell cycle, which points out meaningful directions for future mechanism research.

Based on the clinical significance and functions of TARDBP, we further focused on the DEcircRNAs acting on TARDBP. Among the RBP-circRNA regulatory network, hsa_circ_0000520 and hsa_circ_0004913, both binding to TARDBP, were extracted to generate a hub network. However, most reports about hsa_circ_0000520 are related to gastric cancer [31]. The expression of hsa_circ_0000520 was not significantly different between HCC and adjacent nontumorous tissues [8]. As for hsa_circ_0004913, it recovers the expression of INPPL1 repressed by miR-184 [9] and previous study proved that over expressing INPPL1 or miR-184 silencing inhibits HCC proliferation [32], which indicates that hsa_circ_0004913 may exert an anticarcinogenic effect on HCC. Our study also demonstrated that hsa_circ_0004913 could bind to TARDBP in HCC cells. Combined with the reported results and our findings, we assumed that overexpression of hsa_circ_0004913 may act as sponge of TARDBP and attenuate its effect on the carcinogenesis of HCC, but the specific mechanism still needs to be explored in future study.

In conclusion, we identified some DERBPs interacting with circRNAs and generated RBP-circRNA regulatory networks for HCC. Among the DERBPs, high TARDBP expression was corelated with high grade, advanced stage and low macrophage fraction of HCC. We also constructed the hub RBP-circRNA network based on TARDBP and confirmed that hsa_circ_0004913 could bind to TARDBP, which may provide new clues for HCC mechanism study. However, there are also some limitations in our study. First, we only used TCGA, ICGC and CPTAC projects for analysis and little data resulted in only one RBP with prognostic significance, which may lead to the loss of some potential functional RBPs. Second, we didn’t classify samples according to the etiology and these identified circRNAs and RBPs may not be representative in HCC with different etiologies. Moreover, the number of HCC cases with circRNA data included in this study is relatively small. Due to our current lack of HCC samples and no survival information of HCC with circRNA expression profiles in GEO, we could not verify the expression and assess the prognostic value of circRNAs for HCC. In summary, our results indicated that some RBP-circRNA networks take a potential part in the pathogenesis of HCC and provide a new perspective for further mechanism study and biomarker development of HCC.

## MATERIALS AND METHODS

### Data collection

The paired HCC and non-tumor tissues with circRNA microarray data were retrieved from GSE78520 (3 pairs), GSE97332 (7 pairs) and GSE94508 (5 pairs) in Gene Expression Omnibus (GEO) database (http://www.ncbi.nlm.nih.gov/gds/). For RNA sequence data, 403 cases (containing 199 pairs of HCC and non-tumor tissues) and 392 cases (containing 50 pairs) of transcriptome profile were respectively downloaded from ICGC data portal (https://dcc.icgc.org/) and TCGA data portal (https://tcga-data.nci.nih.gov/tcga/). For mRNA microarray data, 435 cases (containing 214 pairs of HCC and non-tumor tissues) were obtained from GSE14520 in Gene Expression Omnibus (GEO) database (http://www.ncbi.nlm.nih.gov/gds/). For proteomics data, 150 paired HCC and non-tumor cases were obtained from CPTAC (https://proteomics.cancer.gov/programs/cptac). Criteria for study inclusion were: 1) The disease was diagnosed as HCC; 2) HCC caused by different etiologies was acceptable; 3) The HCC cases with RNA sequence or proteomics data had complete expression profile; 4) The corresponding overall survival (OS) time were more than one month.

### Identification of DEcircRNA and DERBPs

Our previous study has mentioned the method to identify DEcircRNA [33]. Briefly, based on |log2FC| > 2 and the significance score < 0.01, Robust rank aggregation method was utilized to detect DEcircRNAs from different datasets. The RBPs potentially binding to circRNAs were predicted by Circular RNA Interactome (Circinteractome, https://circinteractome.nia.nih.gov/) based on CLIP data sets. Next, the protein or mRNA expression level of RBPs were compared between paired non-tumor and tumor cases from CPTAC, TCGA or ICGC projects by Mann-Whitney-Wilcoxon Test, respectively. Those common DERBPs in both protein and mRNA level were screened out for further survival analysis.

### Immune cell fractions analysis

Differing from CIBERSORT or xCELL application, Estimate the Proportion of Immune and Cancer cells (EPIC) method generates an absolute score representing the cell fraction [34]. The fractions of cancer associated fibroblasts (CAFs), B cells, endothelial cells, CD4 T cells, macrophage, and CD8 T cells in HCC were evaluated by EPIC (http://epic.gfellerlab.org/). The differences of cell fractions between TARDBP low- and high-expression groups were also evaluated.

### Gene set enrichment analysis (GSEA)

The HCC cases from TCGA and ICGC were respectively divided into TARDBP low- and high-expression groups. Comparison of the potential biological pathways between the two groups was performed by GSEA (http://software.broadinstitute.org/gsea/index.jsp). The reference gene set was the annotated genomic list c2.cp.kegg. V5.2.symbols.gmt. And the cut-off threshold was FDR < 0. 25 and nominal *P* < 0. 01. The gene sets owning top five standardized enrichment score (NES) in two groups were selected for visualization.

### Quantitative reverse transcription polymerase reaction (qRT-PCR) analysis

Human HCC cell lines MHCC97H, HCCLM3 and immortalized human hepatocyte cell line MIHAwere cultured in Dulbecco’s Modified Eagle’s Medium (DMEM) (pH 7.4) supplemented with 10% (v/v) fetal bovine serum (Gibco). TRIzol reagent (Invitrogen) was used for isolating total RNA from cells and the Prime Script RT reagent kit (Takara Bio, Shiga, Japan) was utilized to synthesize cDNA. qRT-PCR detection was performed through real-time detection system (Roche LightCycler 480, Switzerland) by using the SYBR^®^ Premix Ex Taq™ (Takara). The primer sequences for detection were provided in Supplementary Table 3. GAPDH served as an internal standard control. 2^-ΔΔCt^ method was used to determine the level of gene expression.

### RNA immunoprecipitation (RIP)

Magna RIP Kit (Millipore, Germany) was utilized for RIP assay. In short, magnetic beads were mixed with 5μg anti-rabbit IgG (Millipore, Germany) or anti-TARDBP (#3448, Cell Signaling Technology, MA, USA) and then subjected to the cell lysates. The interested RNAs were eluted from immunoprecipitated complex after proteinase K treatment and then purified for further qRT-PCR analysis.

### RNA pull-down

Pierce Magnetic RNA-Protein Pull-Down Kit (Pierce Biotechnology, USA) was utilized for RNA pull-down assay. The biotinylated DNA probe complementary to hsa_circ_0004913 or negative control probe was incubated with streptavidin-coated magnetic beads at 26°C for 30min to generate probe-coated magnetic beads. 30ul of cell lysate were used for the preparation master mix of RNA-protein binding reaction and part of cell lysate were aliquoted for input. The mix was then incubated with probe-coated beads at 4°C for 30 min. After washing and elution of RNA-binding protein complexes, the proteins in the pull-down materials were analyzed by Western blot.

### Statistical analysis

The differences of OS between HCC cases with low- and high-expression of RBP were accessed by log-rank test and Kaplan-Meier survival analysis. The univariate and multivariate survival analyses were used for the identification of prognostic factors. Mann-Whitney-Wilcoxon Test was used to evaluate the differences of RBP expression among each clinicopathological traits. Data differences between in-vitro experimental groups were analyzed by one-way analysis of variance (ANOVA) or Student’s *t*-test. All tests were analyzed via R software version 3.4.2 with statistically significance of *P* < 0.05.

## Supplementary Materials

Supplementary Figures

Supplementary Tables
