# Supplementary material for: Identification of RNA binding protein interacting with circular RNA and hub candidate network for hepatocellular carcinoma
Source: Aging (Albany NY). 2021 Jun 16;13(12):16124–43. doi: 10.18632/aging.203139 (PMC8266373; doi:10.18632/aging.203139)
Supplement: Supplementary Figures [file aging-13-203139-s001.pdf]

SUPPLEMENTARY FIGURES

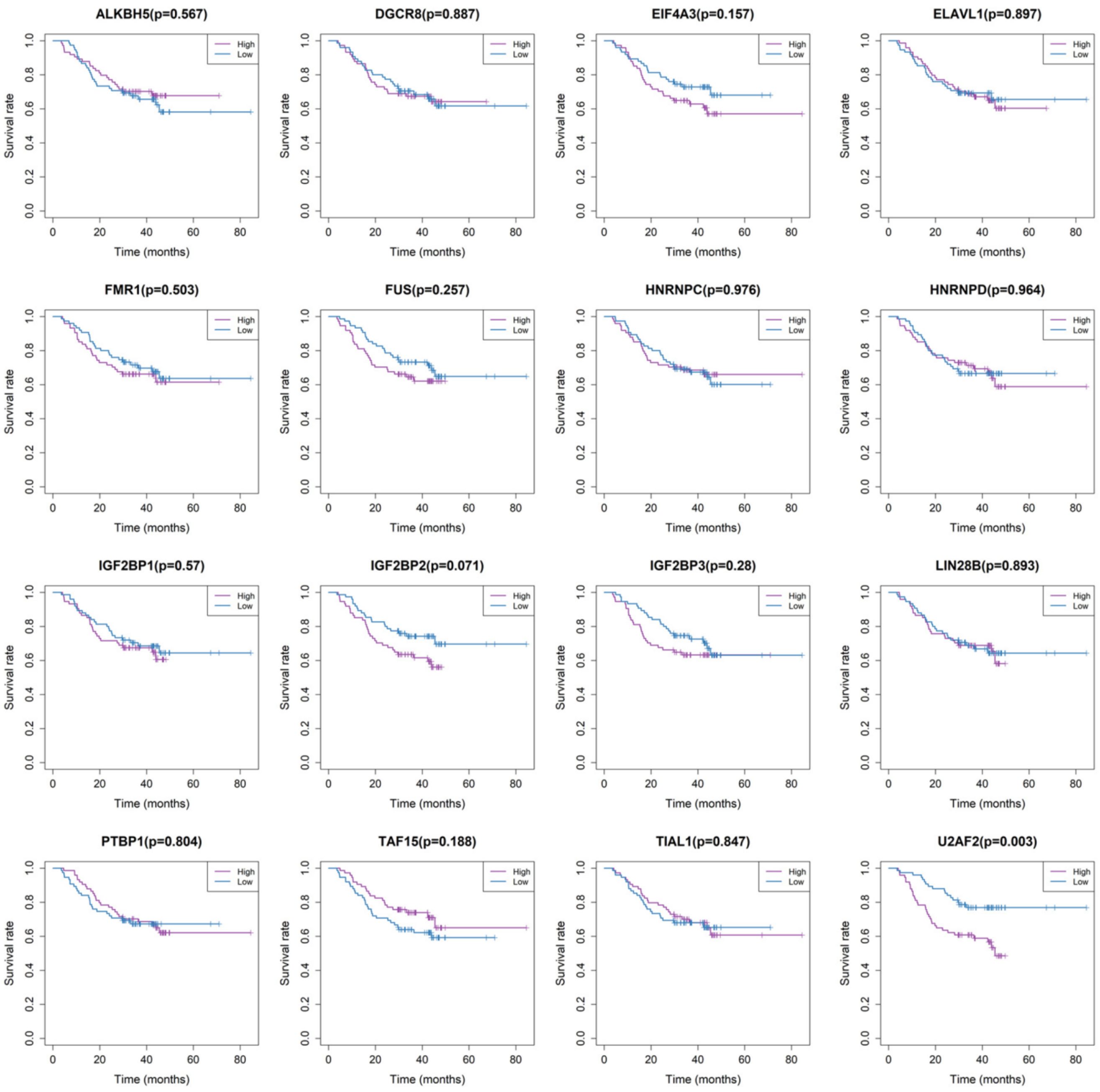

Supplementary Figure 1. Survival analysis of HCC cases with TARDBP low- and high-expression in CPTAC.

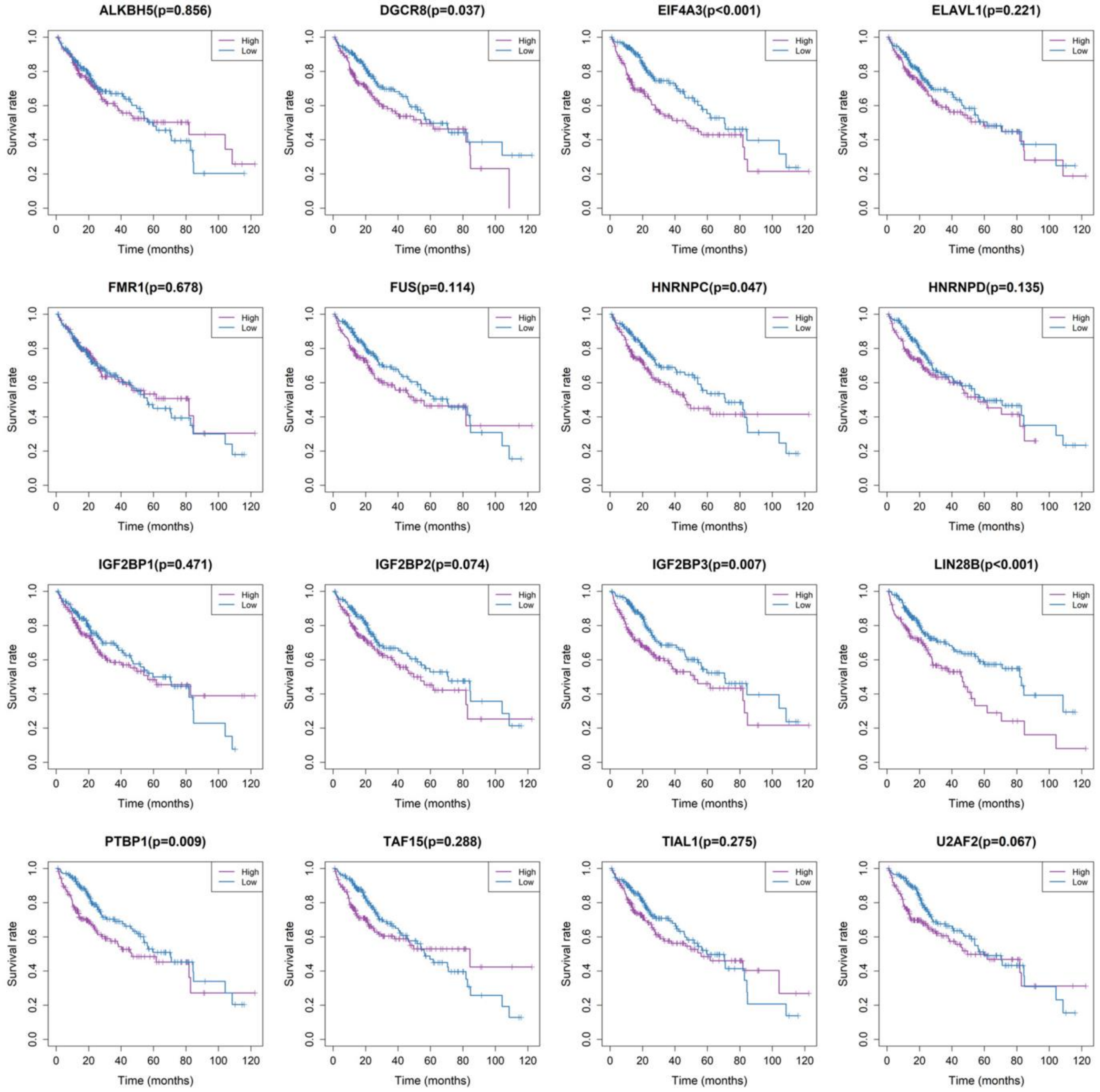

Supplementary Figure 2. Survival analysis of HCC cases with TARDBP low- and high-expression in TCGA.

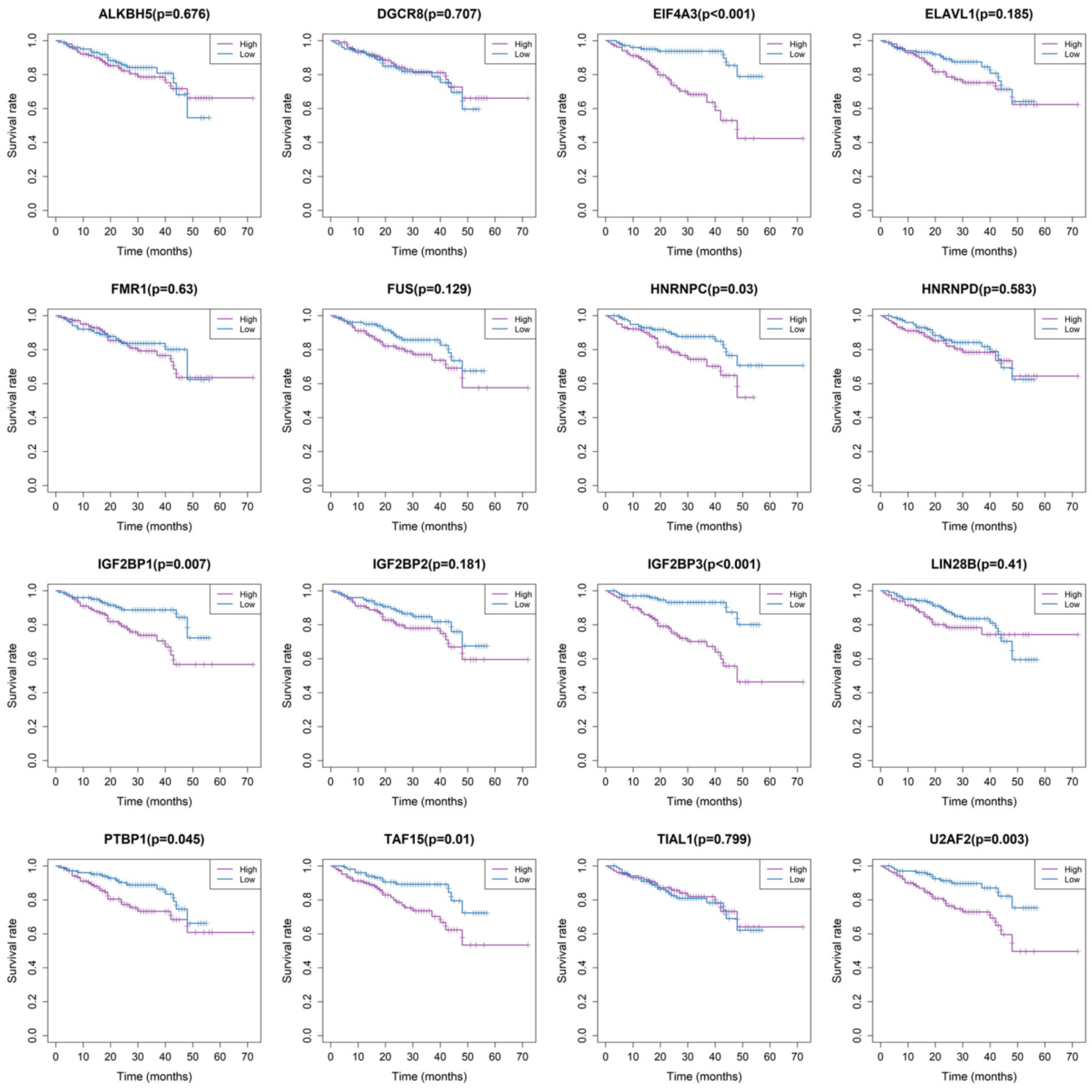

Supplementary Figure 3. Survival analysis of HCC cases with TARDBP low- and high-expression in ICGC.

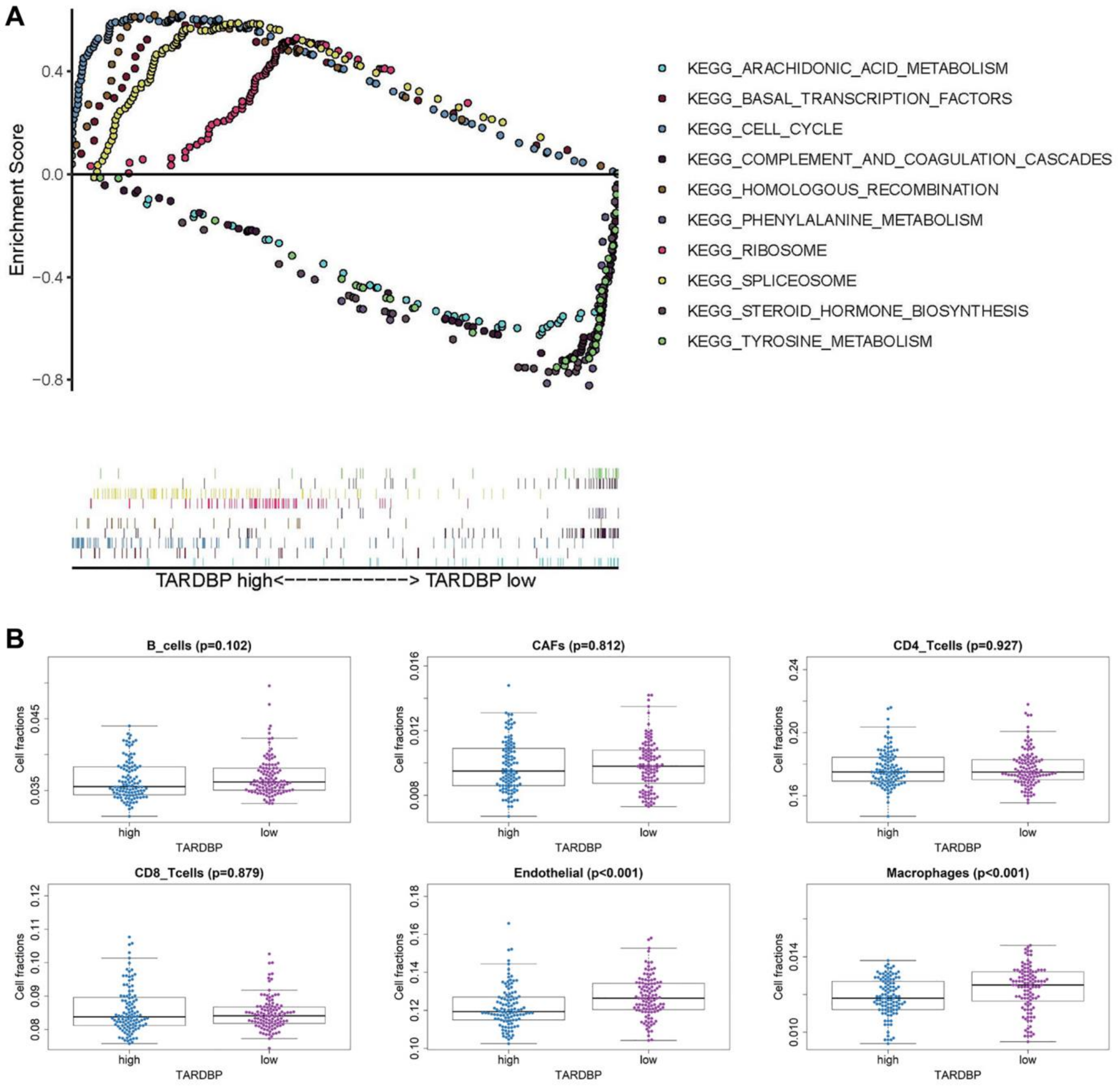

Supplementary Figure 4. GSEA and immune cell fractions analysis of HCC cases with TARDBP low- and high-expression in GSE14520.
