# Supplementary material for: Identification of RNA binding protein interacting with circular RNA and hub candidate network for hepatocellular carcinoma
Source: Aging (Albany NY). 2021 Jun 16;13(12):16124–43. doi: 10.18632/aging.203139 (PMC8266373; doi:10.18632/aging.203139)
Supplement: Supplementary Tables [file aging-13-203139-s002.pdf]

SUPPLEMENTARY TABLES

Supplementary Table 1. 22 DEcircRNAs obtained from 15 paired HCC and non-tumor cases in GEO database.

circRNA	log FC	Significance score
hsa_circ_0067934	2.368467	1.09E-06
hsa_circ_0072088	2.762729	1.61E-05
hsa_circ_0000520	2.41909	2.86E-05
hsa_circ_0006608	2.291249	8.74E-05
hsa_circ_0001955	2.335479	0.000114
hsa_circ_0003763	2.150986	0.000307
hsa_circ_0008274	2.060151	0.000349
hsa_circ_0011385	2.005072	0.000676
hsa_circ_0004913	–4.08705	1.36E-07
hsa_circ_0091570	–3.78638	1.79E-06
hsa_circ_0002980	–3.43061	1.79E-06
hsa_circ_0000098	–3.20732	4.02E-06
hsa_circ_0006302	–2.39652	3.61E-05
hsa_circ_0006168	–2.32895	3.61E-05
hsa_circ_0059369	–2.43762	5.40E-05
hsa_circ_0058493	–2.12698	5.40E-05
hsa_circ_0032683	–2.25806	6.42E-05
hsa_circ_0013339	–2.10431	8.74E-05
hsa_circ_0003258	–2.84322	0.0001
hsa_circ_0027364	–2.1013	0.000129
hsa_circ_0003570	–2.16794	0.006012
hsa_circ_0007456	–2.02803	0.008014

Supplementary Table 2. RBPs bound to DEcircRNAs predicted by Circinteractome.

RBP	circRNA
EIF4A3	hsa_circ_0067934
HNRNPC	hsa_circ_0067934
EIF4A3	hsa_circ_0072088
FUS	hsa_circ_0072088
HNRNPC	hsa_circ_0072088
ELAVL1	hsa_circ_0072088
TIAL1	hsa_circ_0072088
U2AF2	hsa_circ_0072088
AGO1	hsa_circ_0000520
AGO2	hsa_circ_0000520
AGO3	hsa_circ_0000520
ALKBH5	hsa_circ_0000520
HNRNPD	hsa_circ_0000520
NCBP3	hsa_circ_0000520

RTCB	hsa_circ_0000520
DGCR8	hsa_circ_0000520
EIF4A3	hsa_circ_0000520
FMR1	hsa_circ_0000520
FUS	hsa_circ_0000520
ELAVL1	hsa_circ_0000520
LIN28A	hsa_circ_0000520
LIN28B	hsa_circ_0000520
TAF15	hsa_circ_0000520
TARDBP	hsa_circ_0000520
TNRC6A	hsa_circ_0000520
ZC3H7B	hsa_circ_0000520
EIF4A3	hsa_circ_0006608
HNRNPC	hsa_circ_0006608
ELAVL1	hsa_circ_0006608
PTBP1	hsa_circ_0006608
AGO2	hsa_circ_0001955
EIF4A3	hsa_circ_0001955
U2AF2	hsa_circ_0001955
EIF4A3	hsa_circ_0003763
TIAL1	hsa_circ_0003763
AGO2	hsa_circ_0008274
DGCR8	hsa_circ_0008274
EIF4A3	hsa_circ_0008274
U2AF2	hsa_circ_0008274
AGO2	hsa_circ_0011385
DGCR8	hsa_circ_0011385
EIF4A3	hsa_circ_0011385
ELAVL1	hsa_circ_0011385
EIF4A3	hsa_circ_0004913
FUS	hsa_circ_0004913
LIN28A	hsa_circ_0004913
TARDBP	hsa_circ_0004913
U2AF2	hsa_circ_0004913
AGO2	hsa_circ_0091570
EIF4A3	hsa_circ_0091570
EIF4A3	hsa_circ_0002980
PTBP1	hsa_circ_0002980
EIF4A3	hsa_circ_0000098
IGF2BP2	hsa_circ_0000098
PTBP1	hsa_circ_0000098
U2AF2	hsa_circ_0000098
AGO2	hsa_circ_0006302
EIF4A3	hsa_circ_0006302
U2AF2	hsa_circ_0006302
EIF4A3	hsa_circ_0006168
FUS	hsa_circ_0006168
EIF4A3	hsa_circ_0059369

U2AF2	hsa_circ_0059369
AGO2	hsa_circ_0058493
DGCR8	hsa_circ_0058493
EIF4A3	hsa_circ_0058493
HNRNPC	hsa_circ_0058493
QKI	hsa_circ_0058493
EIF4A3	hsa_circ_0032683
EIF4A3	hsa_circ_0013339
ELAVL1	hsa_circ_0013339
IGF2BP2	hsa_circ_0013339
PTBP1	hsa_circ_0013339
U2AF2	hsa_circ_0013339
AGO2	hsa_circ_0003258
EIF4A3	hsa_circ_0003258
FMR1	hsa_circ_0003258
IGF2BP1	hsa_circ_0003258
IGF2BP3	hsa_circ_0003258
U2AF2	hsa_circ_0003258
EIF4A3	hsa_circ_0027364
FUS	hsa_circ_0027364
AGO2	hsa_circ_0003570
DGCR8	hsa_circ_0003570
EIF4A3	hsa_circ_0003570
FUS	hsa_circ_0003570
PTBP1	hsa_circ_0003570
DGCR8	hsa_circ_0007456
EIF4A3	hsa_circ_0007456
ELAVL1	hsa_circ_0007456

Supplementary Table 3. Primers for qRT-PCR.

Gene	Sense primer (5′ to 3′)	Antisense primer (5′ to 3′)
hsa_circ_0004913	TGACATGGGCGTGGCT	CCGGACGGACTGTTGTG
GAPDH	TGTGTCCGTCGTGGATCTGA	TTCGTGTTGAAGTCGCAGGAG
